# Influence of Nordic Walking Training on Vitamin D Level in the Blood and Quality of Life among Women Aged 65–74

**DOI:** 10.3390/healthcare9091146

**Published:** 2021-09-02

**Authors:** Szymon Podsiadło, Agnieszka Skiba, Anna Kałuża, Bartłomiej Ptaszek, Joanna Stożek, Amadeusz Skiba, Anna Marchewka

**Affiliations:** 1 Institute of Clinical Rehabilitation, University of Physical Education in Krakow, 31-571 Kraków, Poland; agnieszka.skiba@awf.krakow.pl (A.S.); ann.kaluza@wp.pl (A.K.); joanna.stozek@awf.krakow.pl (J.S.); amadeusz.skiba@awf.krakow.pl (A.S.); anna.marchewka@awf.krakow.pl (A.M.); 2 Institute of Applied Sciences, University of Physical Education in Krakow, 31-571 Kraków, Poland; bartlomiej.ptaszek@awf.krakow.pl

**Keywords:** old age, Nordic walking, physical activity, vitamin D, quality of life

## Abstract

Introduction. Demographic forecasts indicate the progressive aging process of societies in all countries worldwide. The extension of life span may be accompanied by deterioration of its quality resulting from a decrease in physical activity, mental or even social performance, and a deficit in certain chemical compounds responsible for proper functioning of the body. Aim. The aim of the study was to evaluate the influence of a 12-week Nordic walking (NW) training intervention on the level of vitamin D in the blood and quality of life among women aged 65–74 years. Materials and methods. The study comprised 37 women aged 65–74 (x = 68.08, SD = 4.2). The subjects were randomly assigned to 2 groups: experimental group (NW), which consisted of 20 women who underwent an intervention in the form of Nordic walking training for 12 weeks, and the control group (C), including 17 women who underwent observation. In the experimental group, training sessions were held 3 times a week for 1 h. At that time, the C group was not subject to any intervention. The SF−36 questionnaire was used to measure quality of life. Vitamin D was assessed based on the results of biochemical blood tests. The analysed parameters were assessed twice-before and after the completed intervention or observation. Results. Comparison of the results regarding trials 1 and 2 allowed to note statistically significant improvement in quality of life for all health components and factors in the NW group. Analysis of vitamin D levels demonstrated a statistically significant increase in the NW group. In group C, no significant changes in the analysed parameters were observed. Conclusions. Regularly undertaking Nordic walking training significantly influences the improvement of self-evaluation regarding the components of physical and mental health, as well as the concentration of vitamin D in women aged 65–74 years.

## 1. Introduction

Involutionary processes in the body may adversely affect the quality of life among older adults. One of the methods to solve the problem of an aging society is activity aimed at maintaining or improving physical health and quality of life of seniors [1,2]. Therefore, it is important to identify factors having an influence on the quality of life among older adults [3]. The social dimension regarding quality of life of seniors consists of several issues related to socio-economic, medical-biological, and psychological spheres, which require interdisciplinary research concerning the biopsychosocial approach [4]. Quality of life assessment is subjective and based on a personal perception of one’s own health in the area of physical, mental, and social functioning. Self-evaluation of health among older adults is greatly influenced by the perception of their own functional fitness, guaranteeing self-sufficiency and independence in carrying out activities of daily living. Inability to meet basic needs in the area of personal hygiene or control of physiological needs may lead to deterioration in mental health and, consequently, to the development of anxiety and depressive disorders [5,6]. Some authors cite an appropriate level of physical fitness as the main factor responsible for quality of life among older people, which allows them to function independently and autonomously in various areas of life, both within their families and society [7,8].

Today, vitamin D deficiency is one of the major public health problems worldwide, particularly affecting older adults [9,10]. As a result of the body’s aging and a decrease in the efficiency of the work of many organs, concomitant diseases and the associated large amount of medications taken, the natural ability of the body not only to synthesise cholecalciferol (an inactive form of vitamin D), but also to convert to metabolically active forms, decreases [11]. As shown in epidemiological studies conducted amongst 18 countries and at various latitudes, low vitamin D levels in postmenopausal women were found for nearly all of the studied locations. Overall, 64% of the respondents achieved a level below the generally accepted standards. There are also a number of reports in which the effects are shown of low vitamin D levels on the increased risk of cardiovascular, nervous, endocrine, muscular, and skeletal diseases in older adults [12]. Vitamin D deficiency in older adults is also influenced by gradually developing motor disability, which reduces the time spent outdoors, and as a result, being exposed to UV rays [13,14].

Both of the above-mentioned phenomena allow to suggest that in addition to further concern for extending life expectancy, our actions should be directed towards the implementation of the process of successful aging, i.e., reaching old age with a low risk of diseases and maintaining an appropriate level of physical and mental fitness. One of the forms of physical activity that can contribute to the successful aging process is Nordic walking [15,16]. Such a type of exercise is becoming increasingly popular among older adults, and is an important point of interest for researchers from around the world.

The aim of the study was to investigate the influence of a 3-month Nordic walking training intervention on vitamin D levels and quality of life among women aged 65–74.

## 2. Materials and Methods

### 2.1. Ethical Approval

The studies (prospective, randomized, controlled) were carried out at the Bronisław Czech University of Physical Education in Krakow. The first participant was registered in January and the last in March. The intervention and observation lasted from April to June. The study was approved by the Bioethical Commission of the Regional Medical Chamber in Krakow (number: 237/KBL/OIL/2018 of 11 December 2018). All the procedures complied with the Helsinki Declaration. Informed consent was obtained from all participants.

### 2.2. Characteristics of the Study Group

The study group comprised 37 women aged 65–74 (x = 68.08, SD = 4.82). The inclusion criteria for the study were female gender, age between 65 and 74 years, no Nordic walking training experience in the past, a medical certificate confirming that there were no contraindications for participation in the training, and the respondent’s voluntary consent to be included in the project. The exclusion criteria were unregulated cardiovascular diseases, diabetes, vitamin D supplementation, a period of less than 5 years from completing oncological treatment, mental disorders and/or the inability to move independently. In order to exclude other factors that could influence the studied variables, the participants could not take part in other forms of training during the research project. Women qualified for the study were randomly assigned to one of 2 groups. The group that underwent the intervention in the form of Nordic walking training (NW) consisted of 20 women (mean age x = 68.77, SD = 5.42), while the control group (C) included 17 women (mean age x = 67.38, SD = 4.21) not participating in training.

### 2.3. Research Methods

Participants from both groups were tested twice. The first trial was performed before the intervention in the experimental group, while the second was carried out after three months.

### 2.4. Determination of Vitamin D Levels

A qualified laboratory diagnostician, under the supervision of a physician, drew blood from the elbow, cephalic, or median vein. Blood was collected from the subjects during morning hours and in fasting state, according to the guidelines proposed by the Blood Physiology Laboratory at the University of Physical Education in Kraków. Analysis of blood parameters was performed at an analytical laboratory. The DRG 25 (OH) Vitamin D (total) ELISA Kit was used to determine the level of vitamin D; reading 450 nm (DRG Instruments GmbH, Germany). The electrochemiluminescence (ELISA) method was implemented with the 25 (OH) D (total) competitive test, which is designed to assess the level of vitamin D [ng/mL] in the human body [17].

### 2.5. Evaluating Quality of Life

The SF−36 questionnaire was used to assess quality of life, which consists of 36 questions divided into 8 categories: physical functioning (PF), role-physical (RP), bodily pain (BP), general health (GH), vitality (VT), social functioning (SF), role-emotional (RE), mental health (MH). The sum of points obtained from all 8 elements constitutes the overall health assessment—the higher the score, the better assessed quality of life [18,19].

### 2.6. Description of Intervention

Nordic walking training sessions were held 3 times a week from April to June in a park. Each training unit took place in the morning, lasting 60 min, and was divided into 3 stages: warm-up (10 min), training proper (45 min), and cool-down after activity (5 min). The intensity of the walk proper was set at 60–70% HRmax, which corresponded to 10–12 points on the Borg scale. Prior to beginning the intervention, there were 3 classes to familiarise participants with the correct Nordic walking technique, and a stress test on a mechanical treadmill of increasing intensity was performed to determine individual training loads.

### 2.7. Statistical Analysis

Statistical analysis was performed using Statistica 12 (StatSoft, Tulsa, OK, USA). Descriptive statistics were determined: mean (x), minimum (min), and maximal values (max), as well as standard deviation (SD). Data distribution analysis was performed using parametric tests—the Student’s *t*-test for dependent samples within the group and the same test for independent samples performing comparisons within the groups. The adopted level of statistical significance was *p* ≤ 0.05.

All experiments were performed in accordance with relevant named guidelines and regulations.

## 3. Results

### 3.1. Vitamin D Levels

A statistically significant increase (*p* = 0.000) in the level of vitamin D was noted in the NW group between trial 1 (25.38 ± 10.73 ng/mL) and trial 2 (29.52 ± 9.59 ng/mL) (Table 1). Moreover, in trial 2, a statistically significantly higher level (*p* = 0.040) of vitamin D was observed in the NW group (29.52 ± 9.59 ng/mL) compared to the C group (23.24 ± 8.12 ng/mL) (Table 1) In group C, no statistically significant changes in vitamin D levels were observed after 12 weeks. The studied groups did not differ statistically from each other with regard to baseline values of the examined index.

### 3.2. Quality of Life Assessment

Statistically significant differences were noted for each of the assessed aspects of the SF−36 questionnaire in the NW group between trials 1 and 2 (Table 2). There were also statistically significant differences in each of the examined domains of quality of life in trial 2 between the NW and C group (Table 2). Apart from the assessment of mental health (MH), no significant changes were observed in the control group after the 12-week observation period (Table 2). The study groups, with the exception of social functioning (SF) assessment, did not differ significantly in the baseline levels of the studied traits.

## 4. Discussion

Despite the high popularity of Nordic walking training among seniors, there are still only a few studies on the assessment of its effect on blood parameters. Based on the available literature, it may be concluded that few researchers have addressed this topic. Therefore, an attempt was made to evaluate the effectiveness of a 12-week Nordic walking training programme on vitamin D levels and quality of life among older women. The obtained results allow to indicate that 12 weeks of Nordic walking may have caused changes regarding vitamin D levels D in the blood of the subjects and their quality of life. Below is a brief review of literature concerning the influence of various forms of physical activity on blood vitamin D levels and the assessment of quality of life in older adults. The results of other authors’ research were compared with the results obtained in this study.

Physical activity in the form of Nordic walking training has a positive effect on health, well-being, and quality of life among seniors [20,21]. Aerobic exercise contributes to a number of physiological changes which, as the body adapts to exercise, provide many benefits to the entire human body. Appropriate frequency and duration of training may allow to maintain favourable conditions regarding body functioning for a prolonged period of time [22]. In addition, ‘dosed’ exercise for seniors, regardless of its form, contributes to slowing down the progress of involutional changes and, at the same time, promotes the process of successful aging, i.e., reaching old age with a low risk of developing various types of diseases while maintaining an appropriate level of physical and mental fitness [23,24]. In our study, we noted that Nordic walking training contributes to an increase concerning the level of vitamin D in the blood and improves quality of life among women aged 65–74.

In research carried out to date, the authors have proved the positive effect of Nordic walking training on the processes taking place in an aging body [25]. Improvement in balance, functional fitness, muscle strength, and aerobic endurance have been observed in the older adults population. It has also been confirmed that Nordic walking, thanks to the use of poles, allows to improve gait patterns in older adults. Analysing the impact of using Nordic walking poles on temporal-spatial parameters of walking, i.e., walking speed, stride length, cadence and phases of double and single support, as well as posture-related characters of older people, it has been confirmed that this form of physical activity influences the extension of stride, increases walking speed, and forces the body to maintain more vertical positioning. Additionally, the use of poles while walking reduces fears related to falling [26,27,28,29,30,31,32,33,34].

Knapik et al. (2011) studied 161 subjects above the age of 60 to assess the impact of Nordic walking training on self-evaluation of health. The study included people with at least 2 months of training experience at a frequency of at least 60 min a week, who were assigned to one group declaring the above-mentioned physical activity, and a second group, in which participants were physically active but did not have experience with Nordic walking training. The obtained results of the questionnaire showed a higher level of self-assessment with regard to mental and physical health (tested via the SF−36 questionnaire) in the group performing Nordic walking [35].

The influence of Nordic walking training on the assessment of quality of life was also investigated by Saulicz et al. (2015). In their research, 48 women at a perimenopausal age were qualified for the study. The subjects were randomly assigned to one of 2 groups. In the experimental group, a 4-week Nordic walking training unit was carried out, while in the control group, no intervention was applied. After only 10 training sessions, a significant increase in self-perceived health evaluation was noticed with regard both to physical as well as mental health components [36].

On the other hand, different results were obtained by Lipowski et al. (2019), who did not notice any improvement in self-reported level of health tested via the SF−36 questionnaire among individuals after being subjected to 12 weeks of Nordic walking training. The study group consisted of 52 older women who were assigned to a group with Nordic walking experience (minimum of 4 years), or a beginner group (no previous contact with Nordic walking training). After applying the intervention at a frequency of 3 times a week for 1 h, no improvement in the quality of life results was achieved in any of the study groups. The authors state the reason for this to be the fact that the SF−36 questionnaire relates the quality of life to health, while the study participants were healthy individuals. The authors also suspect that in the case of such a population, extending the training period could have led to obtaining the expected results [37].

The results of our research confirm the reports and assumptions proposed by other authors. The 12-week Nordic walking training unit induced definite improvement in all aspects of the quality of life analysed using the SF−36 questionnaire.

The 12-week intervention in the form of regular Nordic walking training significantly influenced the level of vitamin D in the NW group by increasing its concentration (I: 25.38 ± 10.73 II: 29.52 ± 9.59). In group C, no statistically significant differences in vitamin D levels were noted before or after the trial. As is well-known, cutaneous synthesis of vitamin D depends on the level of sun exposure. Therefore, when assessing the test results, the time during which the intervention was carried out should be taken into account. The study was conducted in the period from April–June, i.e., the months with the best conditions for sunlight exposure in a temperate climate. This relationship would explain the results of research obtained by Pilch et al. (2017), which took place in the months of late autumn—from mid-October to early December—in a temperate climate, i.e., a period with relatively short and little intense sunlight. In the discussed studies, it was shown that the intervention in the form of six-week regular Nordic walking training among postmenopausal women resulted in a significant decrease in vitamin D levels. Pilch et al. suggest that intensified energy expenditure during training could have had an influence on the decrease in vitamin D levels; thus, as a consequence, an increased demand of muscle cells for vitamin D. Therefore, it may be assumed that in our study, a key role for the final vitamin D level in the NW group was played by the time of the year in which the intervention took place [38].

Touvier et al. (2015) showed that people representing high levels of physical activity also exhibited higher concentrations of vitamin D. The highest values of vitamin D were obtained by people declaring activity in open-air conditions, i.e., performing mountain climbing [39]. Interestingly, there are also studies in which it is indicated that there are positive effects of physical activity carried out in closed rooms on the concentration of vitamin D. Wanner et al. (2015) examined 6370 people to study the relationship between the level of physical activity and vitamin D; the results showed a directly proportional relationship (the greater the activity, the higher the vitamin D levels). What is of great importance, the comparison of results between people declaring indoor physical activity and those training outsider showed no significant differences [40]. The lack of variance in the concentration of vitamin D between people practicing outdoor sports and those training indoors was also demonstrated by Aydin et al. (2019) and Villacis et al. (2014), whose study groups comprised professional athletes. In both cases, however, the vast majority of athletes showed vitamin D deficiencies—the great frequency and intensity of training in professional athletes causes the need for vitamin D to increase significantly [41].

In light of the above facts and the results of our research, it may be concluded that physical activity of average intensity, carried out indoors (without direct exposure to sunlight) and outdoors (with sun exposure), is a factor that can positively affect the level of vitamin D. However, taking the results of research conducted by Sela et al. (2012) into account, which clearly show that older people have a better tolerance for physical activity undertaken outdoors, it may be concluded that Nordic walking is one of the best forms of activity supporting the prevention of vitamin D deficiency in older adults [10].

The currently available literature lacks studies on the effects of Nordic walking training on vitamin D levels in older adults. Alarming data on the problem of vitamin D deficiencies in the older population and awareness concerning the importance of its role in the human body indicate the need for further research on effective and appropriate forms of combating these deficiencies. The results obtained in this study and a detailed review of the literature allow us to conclude that regular Nordic walking training has a holistic effect on the quality of life among older people, positively affecting the concentration of vitamin D and all components of health-related self-assessment in women above the age of 65. Therefore, Nordic walking may be considered a greatly effective form activating seniors.

A limitation of the study was the lack of accurate assessment of the sun exposure index in the intervention and control groups. Additionally, during the study, the participants were not controlled in terms of changes in diet or lifestyle, which could have had an influence on the results.

## 5. Conclusions

- The 12-week Nordic walking training programme had a positive effect on the self-reported level of physical and mental health among older women.
- The 12-week Nordic walking intervention increased vitamin D levels in older women.
- Regular physical activity and the prevention of vitamin D deficiency can be key factors in maintaining independence among seniors.
- Nordic walking exercise should be routinely undertaken by older adults.

## Figures and Tables

**Table 1:** Comparison of the mean values of the vitamin D level between NW and C groups.

	NW Group (x ± SD)			C Group (x ± SD)			NW Group vs. C Group	
	Trial 1	Trial 2	*p*(*t*-Test) ≤ 0.05 *	Trial 1	Trial 2	*p*(*t*-Test) ≤ 0.05 *	*p*(*t*-Test) ≤ 0.05 * Trial 1	*p*(*t*-Test) ≤ 0.05 * Trial 2
**Vitamin D level**	25.38 ± 10.73	29.52 ± 9.59	0.000 *	24.06 ± 10.29	23.24 ± 8.12	0.291	0.311	0.040 *

* Significant difference

**Table 2:** Comparison of the mean values of the quality of life between NW and C groups.

Health Components and Factors	NW Group (x ± SD)			C Group (x ± SD)			NW Group vs. C Group	
	Trial 1	Trial 2	*p*(*t*-Test) ≤ 0.05 *	Trial 1	Trial 2	*p*(*t*-Test) ≤ 0.05 *	*p*(*t*-Test) ≤ 0.05 * Trial 1	*p*(*t*-Test) ≤ 0.05 * Trial 2
Physical Component Summary (PCS)	59.25 ± 3.28	72.18 ± 4.23	0.000 *	58.62 ± 4.62	58.39 ± 2.87	0.520	0.833	0.000 *
Physical Functioning (PF)	66.42 ± 4.18	76.45 ± 5.41	0.000 *	63.13 ± 3.76	62.66 ± 3.87	0.503	0.467	0.000 *
Role-physical (RF)	51.82 ± 5.11	64.61 ± 3.28	0.000 *	53.46 ± 5.35	53.76 ± 4.56	0.765	0.376	0.000 *
Bodily Pain (BP)	59.35 ± 4.75	71.52 ± 3.16	0.000 *	62.00 ± 3.87	62.85 ± 5.01	0.381	0.401	0.002 *
General Health (GH)	59.45 ± 3.11	76.15 ± 5.55	0.000 *	55.76 ± 4.99	54.24 ± 4.62	0.053	0.501	0.000 *
Mental Component Summary (MCS)	63.39 ± 5.85	76.14 ± 4.37	0.000 *	61.87 ± 5.63	62.54 ± 3.98	0.353	0.748	0.000 *
Vitality (VT)	60.80 ± 4.16	75.60 ± 3.56	0.000 *	64.88 ± 4,21	64.71 ± 5.33	0.907	0.433	0.000 *
Social Functioning (SF)	75.96 ± 4.77	86.16 ± 5.88	0.000 *	69.49 ± 3.54	67.72 ± 4.21	0.055	0.282	0.000 *
Role-emotional (RE)	62.74 ± 3.87	74.80 ± 4.97	0.025 *	57.59 ± 5.29	59.76 ± 3.66	0.155	0.401	0.000 *
Mental Health (MH)	54.05 ± 5.21	68.00 ± 4.65	0.000 *	52.94 ± 5.47	55.82 ± 4.87	0.027 *	0.318	0.000 *

* Significant difference

## Data Availability

The datasets used and analysed during the current study are available from the corresponding author on reasonable request.
